# Clinical validation of automatic phantom-less quantitative computed tomography for osteoporosis screening: fat region of interest comparison and multidevice validation

**DOI:** 10.1371/journal.pone.0350035

**Published:** 2026-06-25

**Authors:** Yizhang Tong, Rong Gao, Chunxi Liu, Zhiling Ren, Wenjie Fu, Hongyan Yao, Ping Wang, Sheng Zhou

**Affiliations:** 1 First School of Clinical Medicine, Gansu University of Chinese Medicine, Lanzhou, China; 2 Department of Radiology, Gansu Provincial Hospital, Lanzhou, China; Politecnico di Torino, ITALY

## Abstract

**Background:**

Osteoporosis significantly increases fracture risk and healthcare burden. Traditional bone mineral density screening requires dedicated equipment, limiting opportunistic screening. Phantom-less quantitative computed tomography (PL-QCT) enables bone density assessment during routine chest CT examinations but requires comprehensive validation. This study uses fat region of interest (ROI) comparison and multidevice validation to verify the feasibility and accuracy of automatic PL-QCT for osteoporosis screening.

**Methods:**

This prospective study included 252 participants who underwent routine chest CT examinations. An automatic PL-QCT system was used to measure the volumetric bone mineral density at the T11–L1 vertebrae. Clinical validation compared measurements with phantom-based quantitative computed tomography (PB-QCT) using the system’s standard pipeline with automatically selected subcutaneous fat ROIs and, for methodological comparison, manually selected visceral fat ROIs. Diagnostic performance was evaluated via receiver operating characteristic curve analysis with the DeLong test, whereas accuracy was assessed via linear regression, ICC, and Bland‒Altman analysis. Multidevice validation was conducted across five CT scanners.

**Results:**

The automated PL-QCT system under validation (using automatically selected subcutaneous fat ROI) demonstrated excellent diagnostic performance, with an area under the curve (AUC) of 0.962 for osteoporosis and 0.794 for osteopenia, and showed strong correlation with PB-QCT (r = 0.867, ICC = 0.926). In the methodological comparison, the PL-QCT values derived from the manually selected visceral fat ROI showed significantly superior performance to those from the automated system (subcutaneous fat ROI) for the diagnosis of osteopenia (P = 0.029). Multidevice validation demonstrated consistency across the five CT scanners. Gender analysis revealed greater accuracy in females with distinct age-related bone density patterns.

**Conclusions:**

This study validates that the automated PL-QCT system (utilizing its standard pipeline with automatically selected subcutaneous fat ROI) provides a reliable approach for opportunistic osteoporosis screening during routine chest CT examinations. A key methodological finding is that the manually selected visceral fat ROI demonstrated superior performance to the system’s automatically selected subcutaneous fat ROI specifically in osteopenia detection, offering important evidence for optimizing future calibration strategies. The technology’s cross-platform consistency, confirmed by multidevice validation, supports its broad clinical applicability.

## 1 Introduction

Osteoporosis (OP) is a skeletal disorder characterized by a reduction in bone mass and microstructural deterioration, resulting in decreased bone strength and a significantly heightened risk of fracture [1]. Often termed “silent disease,” osteoporosis frequently progresses unnoticed until the occurrence of an first fragility fracture, as patients typically exhibit no obvious symptoms beforehand. These fractures often serve as the first clinical manifestation of osteoporosis and represent its most severe complication, occurring predominantly in the vertebrae and lower limbs. Such fractures not only lead to chronic pain and disability but also may precipitate various psychological issues, markedly diminishing patients’ quality of life and increasing mortality rates within this population [2]. Despite the considerable health burden posed by osteoporosis, the screening rate for this condition remains alarmingly low in China.

Currently, the three primary modalities for measuring bone mineral density (BMD) are dual-energy X-ray absorptiometry (DXA), quantitative computed tomography (QCT), and quantitative ultrasound (QUS) [3]. DXA is widely regarded as the gold standard for clinical BMD assessment because of its established reliability and accuracy [4]. The World Health Organization recommends the use of the T-score from DXA results as the diagnostic threshold for osteoporosis. Specifically, a T-score ≥ −1.0 SD indicates normal bone mass; a T-score between −2.5 SD and −1.0 SD (−2.5 SD < T-score < −1.0 SD) indicates osteopenia; and a T-score ≤ −2.5 SD is diagnostic for osteoporosis [5,6]. However, despite its established role, DXA exhibits inherent limitations that impact diagnostic precision. First, the T-score-based diagnostic framework relies on population-standardized thresholds rather than absolute BMD values, introducing demographic variability. Second, the projectional nature of DXA cannot differentiate osteophytes, facet sclerosis, or abdominal aortic calcification (AAC) from trabecular bone, leading to overestimation of lumbar spine BMD. Third, areal BMD measurements fail to fully capture bone microarchitectural properties critical for mechanical strength, limiting fracture risk prediction accuracy [7,8]. Conversely, QCT is capable of calculating the volumetric bone mineral density (vBMD, mg/cm^3^) of vertebrae, thereby minimizing measurement errors caused by anatomical and pathological factors such as spinal degenerative changes and vascular calcification [9,10]. Additionally, QCT can independently differentiate between cortical and trabecular bone, enabling monitoring of the responses of metabolically active trabecular bone regions to interventions designed to improve BMD [11]. In multiple clinical studies, QCT has demonstrated a significantly higher detection rate for osteoporosis compared to DXA. For instance, research in an elderly Chinese male population reported osteoporosis detection rates of 45.1% with QCT versus only 10.9% with DXA [10]. A similarly significant disparity was observed in postmenopausal women, with detection rates of 46.4% and 17.1% for QCT and DXA, respectively [5]. This systematic under-detection by DXA stems from its technical limitations: as a two-dimensional projection technique, it cannot effectively differentiate between trabecular bone and concurrent spinal degenerative changes (such as osteophytes and sclerosis) or vascular calcification, leading to an overestimation of areal BMD [5,12]. Furthermore, its measurements are influenced by anthropometric factors like body size and composition, which may introduce assessment bias [13,14]. Consequently, QCT demonstrates more reliable diagnostic performance for osteoporosis, proving particularly suitable for assessing bone health in individuals with spinal comorbidities, vascular calcification, or atypical body habitus. Beyond density-based assessments, computational advances have introduced CT-based finite element (FE) modeling as a biomechanically sophisticated approach for fracture risk prediction [15,16]. FE analysis can simulate bone mechanical behavior under physiological loading conditions, providing superior fracture risk stratification compared to density-based methods alone [17,18]. However, the focus of the present study is the opportunistic screening and diagnosis of osteoporosis, rather than individualized fracture risk prediction. The clinical translation of FE analysis requires specialized software, computational resources, and technical expertise, which also limits its widespread application in clinical settings.. In this context, QCT represents a practical balance between technical complexity and clinical feasibility. It enables three-dimensional bone density evaluation while being less technically demanding than finite element analysis, making it an effective and practical solution for opportunistic osteoporosis screening during routine chest or abdominal CT examinations.

QCT can be divided into phantom-based QCT (PB-QCT) and phantom-less QCT (PL-QCT), with the former including both synchronous and asynchronous calibration methods [19]. The calibration process serves as a fundamental component of PB-QCT, aiming to establish a robust conversion algorithm between computed tomography attenuation values (HU, Hounsfield unit) and vBMD. This quantitative transformation requires constructing calibration curves through reference standards of known density [20,21]. PL-QCT employs subcutaneous fat and paravertebral muscle as internal reference points for vBMD measurement, thereby reducing potential instabilities associated with phantom calibration [22–24]. Without the need for phantom calibration, the routine application of chest CT scans permits simultaneous vBMD measurement via PL-QCT during standard chest CT examinations, thus enhancing screening and early detection of osteoporosis [7,25,26]. However, historically, the accuracy of PL-QCT has been suboptimal; studies suggest that the precision of vBMD measurements derived from PB-QCT systems is typically 1.1–2.9 times superior to that of their PL-QCT counterparts, which has posed a significant barrier to the clinical adoption of PL-QCT technologies [27,28]. Furthermore, research conducted by Formica et al. [29] demonstrated that the instability associated with the selection of muscle and fat regions of interest (ROI) is a primary factor influencing the reliability of vBMD measurements in PL-QCT. Previous iterations of PL-QCT relied on manual ROI selection, making the measurement process vulnerable to observer bias, thus compromising the stability and consistency of volume measurements. Liu et al. [27] developed a new automatic PL-QCT system, known as Bone’s FRAX (version: 1.1.1), aimed at achieving precise vBMD and CT value measurements. This software uses subcutaneous fat and paravertebral muscle as self-calibration references and incorporates automatic ROI selection technology for vBMD assessment. Notably, this approach negates the need for a standard phantom and avoids additional radiation exposure during QCT scans, facilitating vBMD measurement via conventional CT images. Building upon the work of Liu et al. [27], this study focuses on addressing two key issues: primarily, through systematic comparison of visceral versus subcutaneous fat ROIs, to determine the optimal calibration reference standard in PL-QCT technology; subsequently, to evaluate the applicability of this system across five different CT devices, verifying its feasibility for clinical. This study aims to provide methodological foundation for the standardized application of PL-QCT technology in opportunistic osteoporosis screening.

## 2 Methods

### 2.1 Population and design

This prospective study was conducted following approval from the Institutional Review Board of Gansu Provincial Hospital (Approval No: 2024−151) and adheres to the principles outlined in the Declaration of Helsinki and good clinical practice standards. Patient recruitment for this study was conducted from March 5, 2024 to June 7, 2024. Written informed consent was obtained from all participating patients. The study cohort comprised patients who underwent routine chest computed tomography (CT) examinations in outpatient, inpatient, or preventive health settings. All imaging procedures were performed by two independent radiologic technologists who were blinded to each other’s findings to mitigate bias. During the routine chest CT scan, a calibrated phantom was positioned for the PB-QCT analysis. Following the completion of this examination, vertebral bone density measurements were obtained for the same patients via the new automatic PL-QCT system, thus ensuring that no additional radiation exposure was incurred. Both the PL-QCT and PB-QCT assessments were completed within one week for each patient.

**Fig 1:**
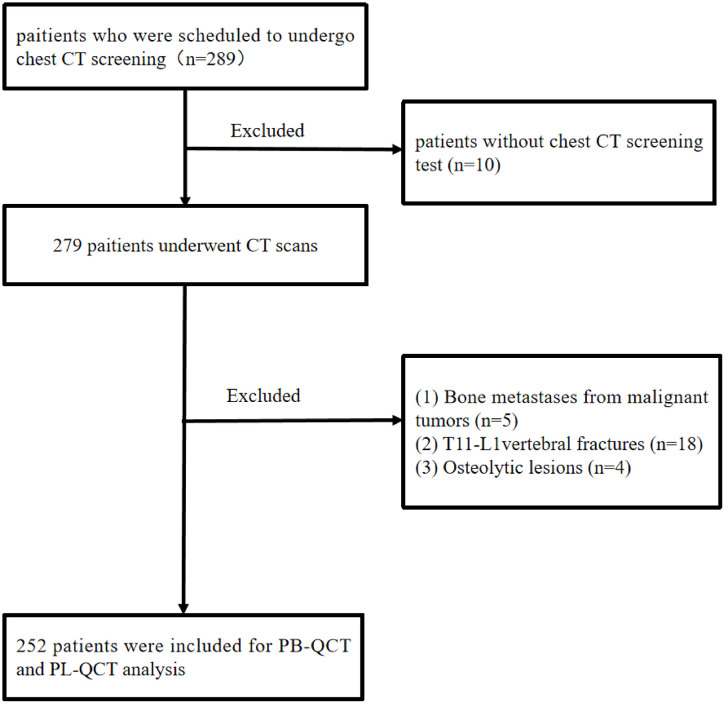
Flowchart of the patient enrollment process.

The exclusion criteria were as follows: 1) patients with a history of spinal fixation surgery; 2) patients presenting with benign or malignant vertebral lesions; 3) patients diagnosed with vertebral compression fractures; 4) patients currently receiving medication known to affect bone metabolism; and 5) patients unable to hold their breath during the chest examination. The screening process is illustrated in Fig 1. Initially, 289 patients were scheduled for the study. During the screening phase, 10 patients without chest CT data were excluded, leaving 279 patients who underwent CT scans. Subsequently, 27 additional patients were excluded: 18 patients were diagnosed with T11-L1 vertebral fractures, 5 patients were identified with bone metastases from malignant tumors, and 4 patients had osteolytic lesions in the vertebrae. Ultimately, 252 patients were included in this study. Basic demographic data, including sex, age, weight, height, and body mass index (BMI), were systematically collected, as detailed in Table 1.

**Table 1:** Basic information of included subjects.

Basic information	Male (n = 146)	Female (n = 106)	Total subjects (n = 252)
Age (years)	50.95 ± 17.39	48.93 ± 16.71	50.10 ± 17.10
Height (m)	1.72 ± 0.05	1.61 ± 0.05	1.68 ± 0.07
Weight (kg)	71.09 ± 11.77	59.39 ± 8.40	66.17 ± 11.96
BMI (kg/m^2^)	23.99 ± 3.80	22.82 ± 3.33	23.50 ± 3.65

Data conform to a normal distribution and are presented as the mean ± standard deviation; BMI, body mass index.

### 2.2 Sample size

This study employed a diagnostic test design. The sample size was calculated a priori using PASS(Power Analysis and Sample Size) Software 2021. Key parameters were set as follows: a significance level (α) of 0.05 for two-tailed tests, statistical power (1-β) of 0.90, and an assumed ratio of osteoporosis patients to normal controls of 1:2. The expected diagnostic performance, measured by the area under the ROC curve (AUC), was set at 0.87, primarily based on the report by Liu et al [27]. and in line with other validation studies [7,30]. The calculation yielded a minimum sample size of 123 subjects, including 41 osteoporosis patients and 82 normal controls. To account for approximately 20% data attrition or inadequate image quality, the target sample size was increased to 150 subjects (50 osteoporosis patients and 100 normal controls). Finally, 252 participants were successfully enrolled, exceeding the minimum theoretical requirement by 105%. The final cohort comprised 27 osteoporosis patients (10.71%), 67 patients with osteopenia (26.56%), and 158 subjects with normal bone mass (62.70%). Although the proportion of osteoporosis patients was lower than initially assumed, the absolute number, combined with the observed high diagnostic performance, still fulfilled the statistical analysis requirements. The actual diagnostic performance of PL-QCT, with AUCs of 0.968 and 0.962 for visceral and subcutaneous fat ROIs, respectively, was higher than the preset value (0.87). The post-hoc power analysis specifically tested the hypothesis that the AUC of PL-QCT was greater than 0.5 (the null hypothesis of random discrimination). Using the preset AUC (0.87) as the effect size and the actual final sample size, the calculated statistical power remained above 90%, meeting the methodological criteria.

### 2.3 Computed tomography

The CT images for this study were acquired from five distinct CT devices: Incisive CT (Philips Healthcare, The Netherlands), IQon Spectral CT (Philips Healthcare, The Netherlands), Definition Flash CT (Siemens Healthineers, Germany), Revolution HD CT (GE Healthcare, USA), and Force CT (Siemens Healthineers, Germany). The detailed CT scanning parameters are specified in Table 2. All imaging procedures adhered strictly to standardized protocols to ensure consistency and reliability in data acquisition.

**Table 2:** CT scanning parameters.

Parameter	Incisive CT	IQon Spectral CT	Definition Flash CT	Force CT	Revolution HD CT
Voltage (kV)	120	120	120	120	120
Tube current(mA)	197	225	300	200	10
SFOV (mm)	500	500	500	500	500
Matrix	512*512	512*512	512*512	512*512	512*512
Table height (cm)	322	118	157	154	165.5
Slice thickness (mm)	1	1	1	1	1.25
Reconstruction kernal	Standard	Standard	Standard	Standard	Standard

SFOV, scan field of view.

### 2.4 PB-QCT analysis

All PB-QCT assessments were performed via asynchronous analysis on a single imaging system utilizing the Mindways QCT standard phantom from Mindways, Inc., to facilitate measurement standardization. Because routine chest CT scans typically cover the T11-L1 region, three specific vertebrae (T11, T12, and L1) were selected for measurement in this study. Compared with extending coverage to L2–L4 vertebrae, this targeted approach enables convenient assessment within standard imaging protocols while minimizing patient radiation exposure, achieving a balance between the diagnostic needs for accurate bone density assessment and adherence to ALARA radiation safety principles [31].

For diagnostic classification, thresholds of ≤80 mg/cm^3^, 80–120 mg/cm^3^, and >120 mg/cm^3^ were used to diagnose osteoporosis, osteopenia, and normal bone mass, respectively. The diagnostic criteria were established on the basis of the ISCD, ACR guidelines [32–34] and the Chinese Expert Consensus on Osteoporosis Diagnosis [35], ensuring their applicability to the Chinese population.

### 2.5 Analysis using the new automatic PL-QCT system

**Fig 2:**
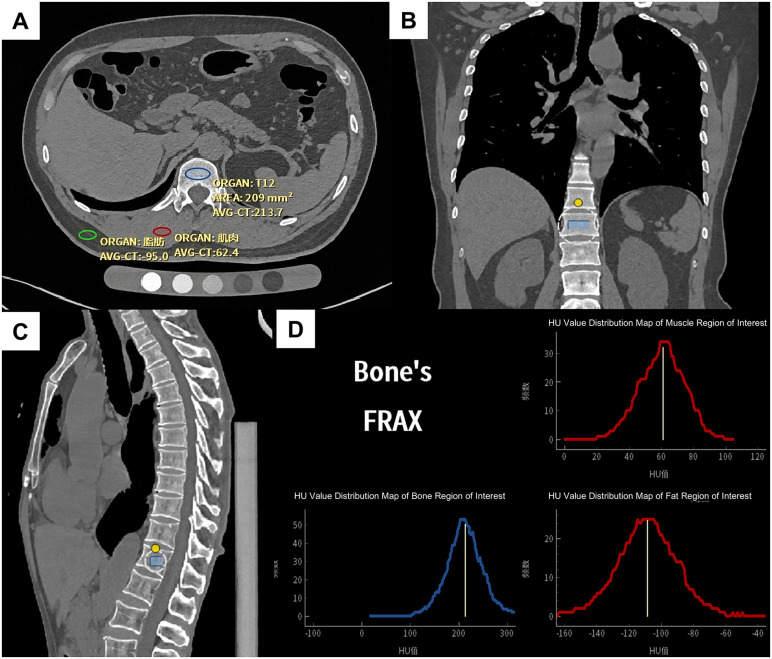
Selection process for the PL-QCT ROI (A-D). (A) Axial CT image showing the vertebral body (blue ellipse) and the reference ROIs for fat (green ellipse) and muscle (red ellipse) tissues. (B) Coronal and (C) Sagittal reconstructed images. (D) Histograms of the HU value distributions for the three ROIs: the top red histogram corresponds to the muscle ROI, the left blue histogram corresponds to the vertebral body ROI, and the right red histogram corresponds to the fat ROI.

In this study, we utilized a new automatic PL-QCT system, Bone’s FRAX (version: 1.1.1), developed by Liu et al. [27], to perform diagnostic analyses on chest CT images obtained from participants. This software employs subcutaneous fat and paravertebral muscle as internal calibration references，with CT value ranges defined as −150 HU to −50 HU for subcutaneous fat and 20 HU to 80 HU for paravertebral muscle, based on clinical expert consensus and tissue-specific HU thresholding. The equivalent density values of the internal reference tissues were predetermined through calibration using a known-density phantom and are embedded as parameters within the software, enabling the construction of a linear HU-BMD calibration curve in conjunction with the automatically acquired average CT value of each ROI. The routine vBMD measurement procedure of this PL-QCT software is as follows: After the CT images from picture archiving and communication system (PACS) were imported through a DICOM interface, the operator precisely positioned a three-dimensional cursor at the geometric center of the T11, T12, and L1 vertebral bodies in the axial, sagittal, and coronal planes. On the basis of this central point, the software automatically generated an elliptical cylindrical VOI with a height of 9 mm and constrained it to the central trabecular bone area via boundary constraint algorithms to avoid interfering structures such as cortical bone and basivertebral venous foramina. The software subsequently automatically selected paravertebral muscle and subcutaneous fat ROIs from the same plane for dual-point calibration (Fig 2) and measured the vBMD for T11–L1 [27]. Furthermore, for methodological comparison, researchers manually delineated visceral fat ROIs. Specifically, the operator manually delineated the ROI in the retroperitoneal or mesenteric area adjacent to the anterior aspect of the vertebral body on the same axial image, carefully avoiding major blood vessels, bowel contours, and mesentery. To ensure a direct and unbiased comparison, the visceral fat ROI was created by copying the automatically selected subcutaneous fat ROI, thereby guaranteeing an identical size and shape, and the CT values within the ROI followed a normal distribution, while the paravertebral muscle ROI remained unchanged. The remaining procedural steps were identical to those described above to derive the vBMD for T11–L1. All measurements were measured three times by a senior radiologist with 15 years of experience in musculoskeletal imaging diagnosis with intervals not exceeding one week, and the average value was calculated to ensure consistency and reliability of the measurements.

## 3 Statistical analysis

### 3.1 Diagnostic performance of visceral and subcutaneous fat-based PL-QCT calibration using PB-QCT as reference

Using PB-QCT measurements as the diagnostic gold standard, with established diagnostic thresholds, participants were classified into three categories: normal, osteopenia, and osteoporosis.. Receiver operating characteristic (ROC) curve analysis was further utilized to evaluate the diagnostic performance of the new automatic PL-QCT system for osteopenia and osteoporosis across these fat ROI configurations. Moreover, we employed the DeLong nonparametric test method to compare the significant differences in the area under the receiver operating characteristic curve (AUC) to evaluate the diagnostic accuracy of different regions of interest (ROIs) from visceral and subcutaneous fat for osteoporosis and osteopenia. Additionally, to explore the influence of sex, we conducted sex-stratified ROC analysis to compare the diagnostic efficacy differences between visceral fat and subcutaneous fat measurement methods in male and female patients.

### 3.2 Accuracy analysis of PL-QCT compared with that of PB-QCT

To evaluate measurement agreement, linear regression analysis, intraclass correlation coefficient (ICC), and Bland-Altman agreement analysis will be employed to assess the agreement between the vBMD measurements from the automated PL-QCT system (using the automatically selected subcutaneous fat ROI) and the PB-QCT gold standard. Subsequently, the same statistical methods will be used to evaluate the agreement between the PL-QCT measurements derived from the manually selected visceral fat ROI and the PB-QCT gold standard. Furthermore, correlation and agreement analyses will be conducted on the PL-QCT measurements obtained from the two ROI selection methods themselves.

### 3.3 Assessing bone density across different CT devices

Previous studies have demonstrated that vertebral marrow attenuation values (CT values) are reliable indicators of the trabecular microstructure and exhibit significant sensitivity and specificity, which are critical for the quantitative assessment of osteoporosis [36,37]. In this investigation, we utilized five distinct models of CT devices for imaging and subsequently conducted scatter plot and linear regression analyses to investigate the relationship between the CT values obtained from these various devices and the corresponding vBMD measurements generated by the new automatic PL-QCT system. We systematically compared the vBMD across five CT devices at different CT value levels and calculated the average percent difference (APD) and coefficient of variation (CV) to quantify the measurement consistency across different CT devices at various density levels and analyze the systematic differences between devices.

### 3.4 Changes in BMD with age

The subjects were divided into five groups by sex and age: 30–39 years, 40–49 years, 50–59 years,60–69 years, and 70–79 years. For each age‒gender group, vBMD was measured via three methods (visceral fat PL-QCT, subcutaneous fat PL-QCT, and PB-QCT). The mean and standard deviation (SD) of each group were calculated, and stratified descriptions and analyses were performed. Changes in vBMD between different age groups are expressed as relative percentages, with difference values calculated between adjacent age groups and between the first and last age groups to quantify the degree of bone loss.

## 4 Results

### 4.1 Basic information

Table 1 summarizes the demographic characteristics of the 252 participants, including sex, age, height, weight, and BMI. Among the male participants (n = 146), the mean age was 50.95 ± 17.3 years, with a mean height of 1.72 ± 0.05 meters, a mean weight of 71.08 ± 11.7 kilograms, and a mean BMI of 23.99 ± 3.8 kg/m^2^. In contrast, the female participants (n = 106) had a mean age of 48.93 ± 16.7 years, a mean height of 1.61 ± 0.05 meters, a mean weight of 59.39 ± 8.3 kilograms, and a mean BMI of 22.82 ± 3.3 kg/m^2^.

### 4.2 Comparison of the diagnostic efficacy of the new automatic PL-QCT system utilizing different fat ROI selections and PB-QCT

**Fig 3:**
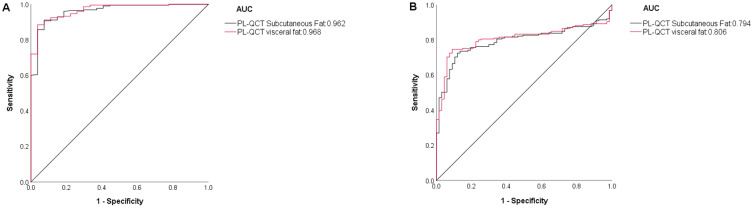
ROC curve of PL-QCT visceral fat and PL-QCT subcutaneous fat. Diagnosis of osteoporosis. (B) Diagnosis of osteopenia. The PL-QCT visceral fat is represented by the red line, and the PL-QCT subcutaneous fat is represented by the black line. PL-QCT, phantom-less quantitative computed tomography; ROC, receiver operating characteristic.

Based on the PB-QCT diagnostic criteria, Table 3 presents the distribution of patients classified as normal, osteopenic, or osteoporotic by each measurement method (PB-QCT, PL-QCT visceral fat, and PL-QCT subcutaneous fat). The PL-QCT visceral and subcutaneous fat measurement methods both demonstrated excellent diagnostic performance for osteoporosis (Fig 3). For the overall population, the PL-QCT visceral fat measurement method had an AUC of 0.968 for osteoporosis diagnosis, with a sensitivity of 88.4%, specificity of 96.3%, and Youden index of 0.85; the PL-QCT subcutaneous fat measurement method had an AUC of 0.962, sensitivity of 90.7%, specificity of 92.6%, and Youden index of 0.83. The DeLong test results (Z = −1.275, P = 0.202, 95% CI: −0.017--0.004) indicated no statistically significant difference in diagnostic ability between the two methods, suggesting that both methods can be used as effective diagnostic tools for osteoporosis in clinical applications. For osteopenia diagnosis, both measurement methods performed slightly less effectively than did osteoporosis diagnosis but still maintained good clinical utility. The AUC values for the PL-QCT visceral and subcutaneous fat measurement methods were 0.806 and 0.794, respectively. The DeLong test revealed that the visceral fat method’s diagnostic capability was significantly superior to that of the subcutaneous fat method (Z = −2.190, P = 0.029, 95% CI: −0.024--0.001). Although the AUC difference(0.012) was small, the difference was statistically significant, suggesting that the visceral fat measurement method may provide more accurate diagnostic information when assessing osteopenia (Table 4).

**Table 3:** Comparison of classification distributions by different measurement methods based on PB-QCT diagnostic criteria.

Method		Normal	Osteopenia	Osteoporosis
PL-QCT visceral fat	Total（n = 252	59.52%(150/252)	29.37%(74/252)	11.11%(28/252)
	Men（n = 146）	58.22%(85/146)	34.25%50/146)	7.53%(11/146)
	Women（n = 106）	61.32%(65/106)	22.64%(24/106)	16.04%(17/106)
PL-QCT Subcutaneous Fat	Total（n = 252）	65.48%(165/252)	26.19%(66/252)	8.33%(21/252)
	Men（n = 146）	65.07%(95/146)	30.14%(44/146)	4.79%(7/146)
	Women（n = 106）	66.04%(70/106)	20.75%(22/106)	13.21%(14/106)
PB-QCT	Total（n = 252）	62.70%(158/252)	26.59(67/252)	10.71%(27/252)
	Men（n = 146）	60.96%(89/146)	32.19%(47/146)	6.85%(10/146)
	Women（n = 106）	65.09(69/106)	18.87%(20/106)	16.04%(17/106)

PB-QCT, phantom-based quantitative computed tomography; PL-QCT, phantom-less quantitative computed tomography.

**Table 4:** Gender subgroup ROC analysis of automatic PL-QCT visceral fat and subcutaneous fat measurements with PB-QCT as the gold standard.

Method		Diagnosis	AUC	Sensitivity(%)	Specificity(%)	Youden index J
PL-QCT visceral fat	Total（n = 252）	Osteoporosis	0.97	88.4	96.3	0.85
		Osteopenia	0.81	74.6	91.0	0.66
	Men（n = 146）	Osteoporosis	0.94	89.7	90.0	0.80
		Osteopenia	0.83	76.8	89.4	0.66
	Women（n = 106）	Osteoporosis	0.99	89.9	100	0.90
		Osteopenia	0.78	72.1	95.0	0.67
PL-QCT Subcutaneous fat	Total（n = 252)	Osteoporosis	0.96	90.7	92.6	0.83
		Osteopenia	0.79	72.4	88.1	0.61
	Men（n = 146)	Osteoporosis	0.93	90.4	90.0	0.80
		Osteopenia	0.81	76.8	85.1	0.62
	Women（n = 106）	Osteoporosis	0.98	94.4	94.1	0.89
		Osteopenia	0.78	72.1	95.0	0.67

PB-QCT, phantom-based quantitative computed tomography; PL-QCT, phantom-less quantitative computed tomography; ROC, receiver operating characteristic; AUC, area under the curve.

The results of the sex stratification analysis revealed that the PL-QCT method demonstrated greater diagnostic accuracy in female subjects than in male subjects. In the female group (n = 106), the PL-QCT visceral fat measurement method achieved an AUC of 0.99 for osteoporosis diagnosis, with a sensitivity of 89.9% and specificity of 100%, whereas in the male group (n = 146), the corresponding metrics were an AUC of 0.94, sensitivity of 89.7%, and specificity of 90%. Similarly, the PL-QCT subcutaneous fat measurement method also showed superior diagnostic performance in females (AUC 0.98) compared with males (AUC 0.93). This finding indicates that this automatic PL-QCT system has greater accuracy in identifying osteoporosis patients in females, effectively avoiding misdiagnosis and unnecessary therapeutic interventions. This sex difference suggests that the PL-QCT may have better clinical application value in female osteoporosis screening.

### 4.3 Accuracy and consistency comparison between PL-QCT and PB-QCT

**Fig 4:**
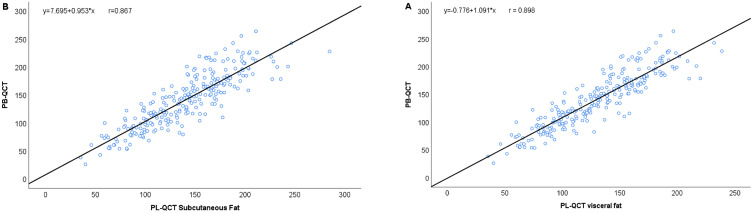
Linear regression equations for PL-QCT visceral fat and PL-QCT subcutaneous fat. Significant positive correlations were found between the PL-QCT of visceral fat, the PL-QCT of subcutaneous fat, and the PB-QCT. (B) Correlation between PL-QCT subcutaneous fat and PB-QCT. (A) Correlation between PL-QCT visceral fat and PB-QCT. PB-QCT, phantom-based quantitative computed tomography; PL-QCT, phantom-less quantitative computed tomography.

**Fig 5:**
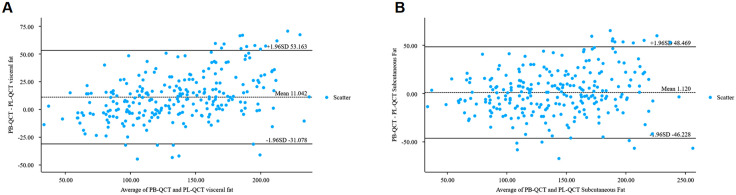
Bland‒Altman agreement analysis. (A) Bland‒Altman plot between PB-QCT and L-QCT visceral fat. (B) Bland‒Altman plot between PB-QCT and PL-QCT subcutaneous fat. The differences in the mean measurement results are shown by the dotted blue lines, and the 95% limits of agreement (mean ± 1.96 SD) are shown by the solid blue lines. The results from the PL-QCT of visceral fat and the PL-QCT of subcutaneous fat and the PB-QCT were in good agreement. PB-QCT, phantom-based quantitative computed tomography; PL-QCT, phantom-less quantitative computed tomography; SD, standard deviation.

Linear regression analysis demonstrated a significant correlation between the measurements obtained from the new automatic PL-QCT system with different fat ROI selections and those from PB-QCT. The regression equation for PL-QCT utilizing the visceral fat ROI was defined as PB-QCT = −0.776 + 1.091PL-QCT (r = 0.898), whereas for PL-QCT using the subcutaneous fat ROI, the equation was defined as PB-QCT = 7.695 + 0.953PL-QCT (r = 0.867) (Fig 4). ICC analysis indicated excellent agreement between the PL-QCT and PB-QCT measurements for both fat ROI selections, with the ICC for visceral fat ROI selection being 0.94 (95% CI: 0.919–0.951) and the ICC for subcutaneous fat ROI selection being 0.93 (95% CI: 0.906–0.942) (Table 5). Bland‒Altman analysis revealed that the mean bias for PL-QCT measurements using the visceral fat ROI was 11.0 mg/cm^3^, with limits of agreement ranging from −31.1 to 53.2 mg/cm^3^, indicating systematic bias between the two measurement methods. In contrast, the mean bias for PL-QCT measurements using the subcutaneous fat ROI was 1.12 mg/cm^3^, with limits of agreement ranging from −46.2 to 48.5 mg/cm^3^. Overall, the majority of data points for both methods fell within the 95% limits of agreement, indicating that the measurements of both methods showed good agreement with the PB-QCT results (Fig 5). Furthermore, a comparative analysis of the agreement between the visceral fat and subcutaneous PL-QCT measurements was performed. The analysis revealed a strong correlation between measurements from the two ROIs (r = 0.983), with a linear regression equation of PL visceral fat = 5.449 + 0.890 PL subcutaneous fat. Bland‒Altman analysis demonstrated that visceral fat ROI-based measurements were systematically greater than subcutaneous fat ROI-based measurements were, with a mean difference of 9.92 mg/cm^3^ and 95% limits of agreement ranging from −7.25 to 27.09 mg/cm^3^. This consistent systematic difference between the two ROI selection approaches may contribute to their varying diagnostic performances at different clinical thresholds.

**Table 5:** ICC analysis results of PB-QCT and automatic PL-QCT measurements.

Method	Intraclass Correlation	95% Confidence Interval
PL-QCT visceral fat	0.937	0.919-0.951
PL-QCT Subcutaneous fat	0.926	0.906-0.942

PB-QCT, phantom-based quantitative computed tomography; PL-QCT, phantom-less quantitative computed tomography; ICC, intraclass correlation coefficient; vBMD, volumetric bone mineral density.

### 4.4 Bone density measurements across different CT devices

**Fig 6:**
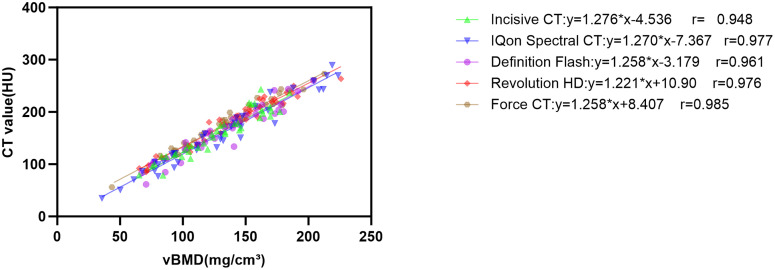
Linear relationship between the CT values and vBMD (mg/cm3) of different CT devices.

This study employed five major CT devices (Incisive CT, IQon Spectral CT, Definition Flash CT, Revolution HD CT, and Force CT) for CT scanning. As shown in Fig 6, significant positive linear correlations were observed between the CT values and vBMD measured by different devices. The scattered distribution demonstrates nearly parallel measurement curves with minimal dispersion, indicating good consistency among devices for BMD quantitative analysis. However, the linear regression equations were not identical, with slight differences in slopes and intercepts reflecting the necessity of device-specific calibration. The quantitative results in Table 6 and S1 Fig demonstrate the systematic differences among the five CT devices at three density levels. At the 100 HU density level, the mean vBMD was 78.85 ± 5.55 mg/cm^3^, with a coefficient of variation (CV) of 7.04%; at the 200 HU density level, the mean vBMD was 158.45 ± 4.66 mg/cm^3^, with a CV of 2.94%; at the 300 HU density level, the mean vBMD was 238.05 ± 3.97 mg/cm^3^, with a CV reduced to 1.67%. The average percentage difference (APD) among the CT devices was greatest at a density of 100 HU, ranging from −7.66% to 7.22%. As the density increased, the interdevice differences gradually decreased, with the APD range narrowing to −2.63% to 1.67% at a density of 300 HU. This finding indicates that in low-density regions (corresponding to low bone mineral density), measurement variations between different CT devices may have more significant impacts on clinical diagnosis.

**Table 6:** Comparative analysis of vBMD measurement variability across five different CT scanners at identical CT values.

CT Value (HU)		Incisive CT	IQon Spectral CT	Definition Flash CT	Revolution HD CT	Force CT	Mean (mg/cm³)	SD (mg/cm³)	CV (%)
100	vBMD(mg/cm³)	81.93	84.54	82.01	72.96	72.81	78.85	5.55	7.04
	APD（%）	3.91	7.22	4.01	−7.47	−7.66	5.55		
200	vBMD(mg/cm³)	160.29	163.28	161.51	154.87	152.30	158.45	4.66	2.94
	APD（%）	1.16	3.05	1.93	−2.26	−3.88	4.66		
300	vBMD(mg/cm³)	238.65	242.02	241.00	236.77	231.79	238.05	3.97	1.67
	APD（%）	0.25	1.67	1.24	−0.54	−2.63	3.97		

vBMD, Volumetric bone mineral density; APD, Average Percent Difference; SD, standard deviation; CV, Coefficient of Variation.

Linear regression analysis revealed strong positive correlations between CT values and vBMD across all the CT devices.

1. Incisive CT: y = 1.276x-4.536, r = 0.948
2. IQon Spectral CT: y = 1.270x-7.367, r = 0.977
3. Definition Flash CT: y = 1.258x-3.179, r = 0.961
4. Revolution HD CT: y = 1.221x + 10.90, r = 0.976
5. Force CT: y = 1.258x + 8.407, r = 0.985

The x-axis represents vBMD (mg/cm^3^), and the y-axis represents CT values (HU).

### 4.5 BMD changes are associated with age for males and females

**Fig 7:**
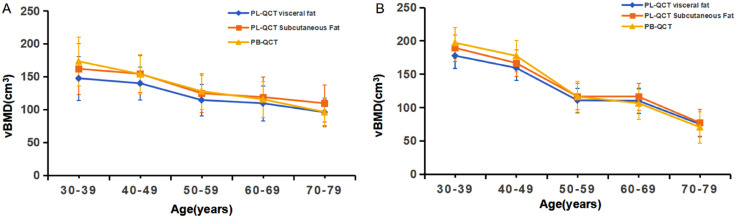
Age-related changes in vBMD across different measurement methods in males and females. The graphs display vBMD measurements (mg/cm^3^) across five age groups (30–39, 40–49, 50–59, 60–69, and 70–79 years) in males (A) and females (B). Three measurement methods are compared: PL-QCT for visceral fat (blue line), PL-QCT for subcutaneous fat (orange line), and PB-QCT (yellow line). PB-QCT, PB-QCT, phantom-based quantitative computed tomography; PL-QCT, phantom-less quantitative computed tomography; vBMD, volumetric bone mineral density.

As shown in Fig 7, vBMD demonstrated age-related decreasing trends in both males (A) and females (B) across five consecutive age groups, with three measurement methods (visceral fat PL-QCT, subcutaneous fat PL-QCT, and PB-QCT) showing similar age-related change patterns. The three measurement methods exhibited high consistency in assessing vBMD across various age and sex groups, with measurement curves showing similar trends. The data revealed that in males, the vBMD measured via PB-QCT gradually decreased from 173.16 mg/cm^3^ in the 30--39 year group to 96.04 mg/cm^3^ in the 70--79 year group; in females, it declined from 197.04 mg/cm^3^ in the 30--39 year group to 70.48 mg/cm^3^ in the 70--79 year group, indicating a more significant decline. In the 30–39- and 40–49-year-old age groups, female vBMD values were on average greater than those of age-matched males, which is consistent with previous research reporting that bone density in young women is generally greater than that in men [38,39]; however, in the 60–69- and 70–79-year-old age groups, male vBMD values exceeded those of females. Females experienced a sharp decline in vBMD after age 40, particularly between 40–49 years (PB-QCT mean value of 177.38 mg/cm^3^ and 50–59 years (116.21 mg/cm^3^), with an approximately 34.5% decline, whereas males showed a gradual decline, with an approximately 17% decrease during the same period. The bone loss rate data indicated that after the age of 50, the annual loss rate in females was approximately 1.6 times greater than that in males (1.97%/year vs 1.24%/year, p < 0.001), resulting in a cumulative vBMD reduction of 58.9% in females from the age of 30--79, compared with 31.5% in males during the same period. These findings have potential clinical implications for sex-specific osteoporosis intervention strategies and screening protocols.

## 5 Discussion

This prospective study evaluated the clinical performance and diagnostic accuracy of an automated PL-QCT system for osteoporosis screening in a cohort of 252 subjects. This study employed a systematic comparison of manually delineated visceral fat versus automatically selected subcutaneous fat ROI selection methods and cross-device validation via five different CT scanner platforms. Comparative validation analysis with PB-QCT confirmed good measurement precision and diagnostic performance of the system, with both ROI methods showing strong correlation (r > 0.86) and high reliability (ICC > 0.92). The DeLong test indicated that the visceral fat ROI method was significantly superior to the subcutaneous fat ROI method in the diagnosis of osteopenia (P = 0.029). Cross-device validation revealed that the system maintained good measurement consistency across five different CT scanner platforms, confirming the stability and broad applicability of the technology. Gender- and age-stratified analyses revealed characteristic patterns of bone density changes across different populations, providing an important reference for developing individualized screening strategies.

Our study results demonstrate that both the PL-QCT visceral and the PL-QCT subcutaneous fat measurement methods have high diagnostic value for osteoporosis and osteopenia. For osteoporosis diagnosis, the two methods performed comparably (AUCs of 0.968 and 0.962, respectively, P = 0.202), both serving as effective clinical diagnostic tools. With respect to osteopenia, the visceral fat measurement method had a statistically significant advantage (AUC 0.806 vs 0.794, P = 0.029). Although the AUC difference was small, the results suggest that the visceral fat measurement method may have better diagnostic value in osteopenia assessment. Prior studies have similarly highlighted that visceral fat ROI selection is associated with enhanced diagnostic efficiency compared with subcutaneous fat ROI selection in the context of osteoporosis and osteopenia [40,41]. Furthermore, adipose components such as marrow fat, visceral adipose tissue (VAT), and adiponectin have been identified as independent predictors of BMD loss, emphasizing the pivotal role of fat composition in the reduction in BMD among older males [40]. Collectively, these observations suggest that selecting visceral fat ROIs may yield more accurate and reliable results in assessing bone health, potentially because of the stronger association between visceral fat and BMD. The differences identified through sex-stratified analysis indicate that the automatic PL-QCT system has greater diagnostic efficacy in the female population, with AUC values of 0.99 and 0.98 for visceral and subcutaneous fat measurement methods, respectively, than those in the male population (AUC values of 0.94 and 0.93, respectively). Epidemiological data show that the prevalence of osteoporosis among postmenopausal women globally is approximately 30% [42,43], representing an important high-risk population. Given the excellent diagnostic performance of PL-QCT in the female population, this method can provide accurate bone density assessment for postmenopausal women, facilitating early diagnosis and treatment monitoring of osteoporosis. These findings provide empirical evidence for selecting appropriate osteoporosis screening strategies for different sex populations.

Tang et al. [7] conducted a comparison between the new automatic PL-QCT system and dual-energy X-ray absorptiometry (DXA), reporting good diagnostic agreement for osteoporosis (AUC = 0.77) and a strong correlation between the system’s measured CT values and BMD (r = 0.85–0.99). Guo et al. [44] developed and validated a semiautomated PL-QCT system based on chest CT for assessing proximal humeral BMD, demonstrating high agreement with the gold-standard phantom-based QCT (R^2^ = 0.97) and acceptable measurement precision (CV = 5.64%–6.17%). In the present study, we compared the the PL-QCT measurements from the two different fat ROI selection methods with conventional asynchronous PB-QCT measurements, and conducted correlation analyses through linear regression, Bland‒Altman, and ICC analyses—key validation metrics commonly employed in clinical diagnostic research. The linear regression analysis revealed strong correlations, with Pearson correlation coefficients (r) ranging from 0.87 to 0.90 between the PL-QCT and PB-QCT measurements for different fat ROI selections. ICC analysis revealed that the ICC for PL-QCT with different fat ROI selections was greater than 0.9, confirming the excellent consistency between PL-QCT and PB-QCT. Although the Bland‒Altman analysis indicated a degree of systematic bias in the PL-QCT results when the visceral fat ROI was used compared with the PB-QCT results, the majority of the data points for both fat ROI selections fell within the 95% limits of agreement. In summary, excellent agreement was observed between PB-QCT measurements and those obtained from both the automated PL-QCT system (using automatically selected subcutaneous fat ROI) and the PL-QCT values derived from the manually selected visceral fat ROI. By further comparing the two fat ROI selection methods, we found that despite the strong correlation between the PL-QCT measurements based on the visceral fat and subcutaneous fat ROIs (r = 0.983), the visceral fat ROI measurements were systematically greater than the subcutaneous fat ROI measurements were (mean difference of 9.922 mg/cm^3^). This systematic difference may explain why the two methods performed similarly in osteoporosis diagnosis, while the visceral fat ROI method demonstrated a significant advantage (P = 0.029) in osteopenia diagnosis. It should be noted that the manual delineation of fat ROIs in this study was performed by experienced operators adhering to standardized protocols, ensuring consistent ROI areas and anatomical levels. We contend that the observed improvement in diagnostic performance is primarily attributable to the inherent properties of visceral fat tissue—such as potentially greater stability of its CT attenuation values or a closer physiological association with bone metabolism—rather than the manual procedure itself. We fully acknowledge, however, that manual delineation remains dependent on operator judgment, which may affect measurement reproducibility and is therefore unsuitable for routine clinical application. Consequently, future research should focus on developing robust algorithms capable of automatically and accurately identifying visceral fat ROIs. Automating this step holds the potential to translate the methodological advantage identified in this study into a fully automated screening tool that retains high diagnostic performance while offering excellent reproducibility and clinical utility.

CT value measurement techniques for vertebral bodies are essential in the assessment of osteoporosis. The CT value represents the average X-ray attenuation of all substances within each voxel, expressed in HU; the magnitude of the CT value directly reflects the density of the tissue [37]. The reliability and accuracy of CT values in measuring bone density and diagnosing osteoporosis have been substantiated in numerous studies [45–48]. For instance, J. Berger-Groch et al. [46] compared the accuracy of HU measurements in CT scans of the lower lumbar vertebrae (L4, L5) and sacrum (S1) for diagnosing osteoporosis and concluded that HU measurements in CT are effective tools for assessing lumbar and sacral bone density. Additionally, Masashi et al. [47] evaluated the relationship between DXA-measured BMD and the HU within the ROI and the volume of interest (VOI) on lumbar CT, revealing a strong correlation between HU values in the VOI and BMD, which can be utilized to predict osteoporosis. Zhang et al. [48] explored the correlation between CT values and BMD in elderly patients with proximal humeral fractures and concluded that proximal humeral CT values represent a rapid and accurate method for assessing BMD in this population. However, some studies have indicated that the current application of CT values for osteoporosis screening lacks sufficient clinical applicability. Due to variations in scanner design and calibration protocols, measurements obtained from different CT devices are not directly comparable. Consequently, values acquired on one machine cannot be generalized to others without device-specific adjustments, which hampers the widespread clinical implementation of this technique [49,50]. In this study, we tested five major CT devices and reported that the linear regression relationships between CT values and corresponding vBMD values were similar but not identical. The statistical results indicated that the coefficient of variation between CT devices was greater at low-density levels but decreased with increasing density. This was confirmed by Zhao et al.’s phantom experiment: the vBMD median of 52.2 mg/cm^3^ in the low-density L1 vertebral region showed significant variation from the calibration value, whereas measurements in the high-density L3 vertebral region were significantly more consistent [51]. We speculate that this is caused by different device parameters and radiation doses across CT devices. To address this issue, we recommend adopting multifaceted coordinated unification measures. First, establishing independent calibration models for different CT scanners can significantly improve the reproducibility of vBMD measurements, with Bodden et al.’s study showing that the relative root mean square difference coefficient can be reduced to 3.7% after calibration [52]. Second, using standardized phantoms such as the European Spine Phantom (ESP) for cross-device verification, with Zhao et al.’s multicenter study validating that this method can effectively correct systematic biases between different brand CT devices, controlling the median difference of L1-3 vBMD within 1.4 mg/cm^3^ [51]. Third, applying automated segmentation and correction technologies based on convolutional neural networks can further enhance consistency, with Sollmann et al.’s research confirming strong correlation (ICC > 0.9) in comparisons between routine CT and dedicated quantitative CT [53]. By combining these strategies, we can effectively reduce systematic differences in bone density measurements between CT devices, thereby improving the reliability of cross-device research and clinical applications.

Having established the technical reliability of our multidevice validation, we further investigated sex-specific bone density patterns through age-stratified analysis, which revealed significant differences in vBMD changes with age. As shown in Fig 7, both male and female vBMD values demonstrated age-related decreasing trends, but their change trajectories exhibited substantial differences. Females maintained relatively high bone density levels from ages 30−49, followed by a sharp decline at approximately age 50 (perimenopause) of approximately 34.5%, whereas males showed a more linear “gradual” bone loss pattern, with only an approximately 17% decline during the same period. This differential change pattern is largely consistent with previous research reports: Cui’s study demonstrated that the peak of bone loss in females occurs between ages 51−55, with a rapid loss period at approximately age 50 rather than uniform changes [54]; Pickhardt’s research confirmed that the average annual loss rate in males is −1.0%, which is lower than that in females, with more gradual changes across different age groups; the rapid bone loss in postmenopausal women is attributed primarily to increased osteoclast activity and accelerated bone resorption caused by the sharp decline in estrogen levels during menopause [55,56]. Our quantitative analysis revealed that the annual bone loss rate in females after age 50 (1.97%/year) was approximately 1.6 times greater than that in age-matched males (1.24%/year) (p < 0.001), which explains the aforementioned high prevalence of postmenopausal osteoporosis in women. Notably, all three methods (visceral fat PL-QCT, subcutaneous fat PL-QCT, and PB-QCT) demonstrated highly consistent BMD‒age trends, further validating the reliability of the measurement methods and the robustness of the obtained data. These findings provide empirical evidence for the development of precision-based osteoporosis prevention and treatment strategies for different sex groups: females may require more frequent bone density monitoring and more aggressive interventions during the perimenopausal period, whereas males, despite having overall slower bone loss rates, still need attention to bone health assessment in populations over 70 years of age, particularly those with other osteoporosis risk factors. Future research should explore in depth the mechanisms linking physiological hormone levels, metabolic status, and sex-related bone loss patterns, as well as their clinical significance.

**Fig 8:**
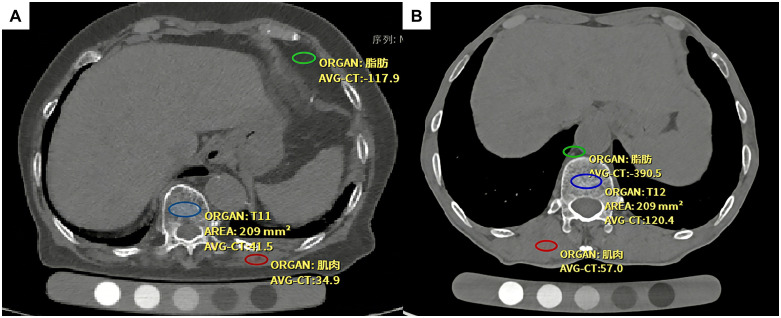
Clinical case comparisons showing how BMI affects the accuracy of PL-QCT measurements. (A) A female aged 86 years, with a BMI of 26.6, had PL-QCT test results of T11-L1 of 42.2 mg/cm^3^, 39.7 mg/cm^3^, and 38.8 mg/cm^3^, and the PB-QCT test results were 26.9 mg/cm^3^, 24.6 mg/cc, and 27.7 mg/cm^3^, respectively. In this elderly obese patient, fatty infiltration of the paravertebral muscles led to reduced muscle density(blue circle), affecting the final measurement results. (B) A male aged 70 years, with a BMI of 18.8, had PL-QCT test results of T11-L1 of 138.1 mg/cm^3^, 120.6 mg/cm^3^, and 109 mg/cm^3^ and PB-QCT test results of 111.2 mg/cm^3^, 92.5 mg/cm^3^, and 87.7 mg/cm^3^, respectively. In this low-BMI patient with a limited fat area at the measurement level, the green circle indicates where the automatic system erroneously includes nonadipose tissue in fat ROI selection, potentially contributing to measurement discrepancies. Blue ellipse, Vertebral body ROI; Green ellipse, Fat ROI; Red ellipse, Muscle ROI PB-QCT, phantom-based quantitative computed tomography; PL-QCT, phantom-less quantitative computed tomography; BMI, body mass index; ROI, region of interest.

Under normal physiological conditions, intramuscular adipose tissue (IMAT) performs multiple physiological functions, including energy storage, provision of metabolic substrates, secretion of adipokines and inflammatory mediators, and modulation of the muscle microenvironment and signaling pathways. However, when fat accumulation exceeds the capacity of the IMAT, localized fat infiltration may occur [57,58]. Research conducted by Kitagawa et al.[59] indicates that Paravertebral fat infiltration is closely associated with obesity, advanced age, and other metabolic diseases. In our analysis of the study results, we observed that older patients with a higher BMI often exhibited reduced density in the paravertebral muscle due to muscle atrophy and fat infiltration, which subsequently impacted the final measurement of vBMD. Conversely, patients with a lower BMI, owing to their reduced body fat content, may have had the system erroneously including nonfat ROIs when selecting fat ROIs, thereby affecting the final vBMD results. We believe that this is one of the key factors contributing to the discrepancies between the measurements obtained from the new automatic PL-QCT system and those from PB-QCT (Fig 8). Yu et al. [60] also supports our viewpoint, indicating that obesity and weight loss exert complex effects on bone density measurements via DXA and QCT, potentially leading to increased measurement errors, although the underlying mechanisms remain unclear. On the basis of these findings, our study demonstrated a strong correlation between PL-QCT and the reference standard PB-QCT. However, compared with previous studies, our correlation coefficient was slightly lower. For comparison, Liu et al. reported a PL-QCT and PB-QCT correlation coefficient of r = 0.99 under single-device conditions [27], whereas Li et al. reported a coefficient of determination R^2^ = 0.96 (P < 0.001) between PL-QCT and PB-QCT for lumbar spine BMD measurements in their study [30]. We attribute this difference primarily to two systematic error sources: BMI-related tissue density variations and CT device parameter differences. Specifically, regarding biological variation, fat infiltration in high-BMI patients and insufficient adipose tissue in low-BMI patients both impact the accuracy of PL-QCT measurements, a phenomenon clearly illustrated in the case study presented in Fig 8. From a technical variation perspective, the interdevice variation coefficient of 7.04% in low-bone density regions constrains the achievement of higher intermethod correlation coefficients. We believe that these systematic errors can theoretically be corrected through appropriate calibrations, including the device-specific calibration measures mentioned earlier and optimized fat ROI selection algorithms tailored to different BMI ranges. Through comprehensive improvements, we are confident that the correlation coefficient between PL-QCT and PB-QCT can be elevated to a more ideal level, better satisfying the requirements of precise clinical bone density measurements.

While our study provides significant insights, its limitations should be carefully considered. First, during the research process, we found that BMI and paraspinal muscle fat infiltration may be potential confounding factors affecting QCT bone density measurements. However, this study did not explore stratified analyses for patients with different BMIs or the quantitative relationship between paraspinal muscle fat infiltration and measurement outcomes. In future research, the extent of the influence of these potential confounding factors on bone density measurement results and their underlying mechanisms need to be systematically evaluated through rigorously designed cohort studies. Second, the study population was primarily recruited from Gansu Province, China, with the majority being Han Chinese, representing a limited geographical region and sample size. Therefore, multiregional studies with larger sample sizes are needed to validate the accuracy and reliability of our findings.

## 6 Conclusion

This study demonstrates that the predominantly automated PL-QCT system, utilizing its standard pipeline with the automatically selected subcutaneous fat ROI, provides a reliable approach for opportunistic osteoporosis screening during routine chest CT examinations. A key methodological finding is that the manually selected visceral fat ROI demonstrated significantly superior performance to the system’s automatically selected subcutaneous fat ROI in osteopenia detection (P = 0.029). This finding provides important evidence for optimizing future calibration strategies. The multi-device validation across five CT scanner platforms further confirms the system’s technical stability and clinical applicability across different healthcare settings, while the phantom-less approach maintains diagnostic accuracy comparable to conventional phantom-based methods yet eliminates the need for external calibration phantoms, thereby simplifying workflows and reducing operational costs. By leveraging existing routine CT examinations without additional radiation exposure or dedicated scanning time, this technology enables efficient population bone health assessment and establishes a solid foundation for integrating automated PL-QCT into clinical workflows to facilitate early osteoporosis detection, though factors such as CT device variability and patient BMI may influence accuracy, as noted in our multidevice validation. Thus, the study results support further clinical evaluation of the automated PL-QCT system in current routine protocols and the exploration of optimized calibration methods based on the visceral fat finding, with these considerations in mind.

## Supporting information

S1 Data Dataset 1. (XLSX)

S2 Data Dataset. (XLSX)

S1 Fig Distribution of vBMD measurements across five CT scanners at different CT value levels. The boxplot shows the pooled distribution of volumetric bone mineral density (vBMD) values measured by the five scanners at 100, 200, and 300 HU. The median and range of measurements increase with higher CT values, while the inter-scanner variability (box height) decreases.
(TIF)
