# Supplementary figures and images for: Clinical validation of automatic phantom-less quantitative computed tomography for osteoporosis screening: fat region of interest comparison and multidevice validation
Source: PLoS One. 2026 Jun 25;21(6):e0350035. doi: 10.1371/journal.pone.0350035 (PMC13298776; doi:10.1371/journal.pone.0350035)

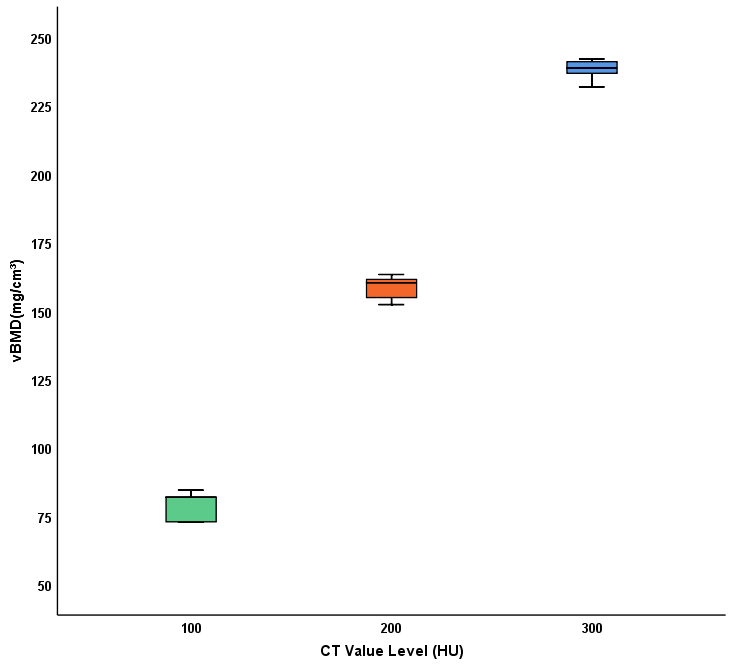

Supplement: S1 Fig — The boxplot shows the pooled distribution of volumetric bone mineral density (vBMD) values measured by the five scanners at 100, 200, and 300 HU. The median and range of measurements increase with higher CT values, while the inter-scanner variability (box height) decreases. (TIF) [file pone.0350035.s003.tif]
